# Real-Time Detection of Urban Atmospheric Micro–Nanoplastics and Their Chemical Mixing State Using Bioaerosol Single-Particle Mass Spectrometry

**DOI:** 10.1021/acs.est.5c06513

**Published:** 2025-09-29

**Authors:** Chongchong Zhang, Yiming Qin, Lei Li, Eleonora Aruffo, Shaoyong Li, Xuan Li, Ning Zhang, Yun Wu, Haiwei Li, Yunjiang Zhang, Yuan Dai, Ming Wang, Xinlei Ge, Ke Li, Wei Du, Chunlei Cheng, Mei Li, Mindong Chen, Junfeng Wang

**Affiliations:** † Jiangsu Key Laboratory of Atmospheric Environment Monitoring and Pollution Control (AEMPC), Collaborative Innovation Center of Atmospheric Environment and Equipment Technology (CIC-AEET), School of Environmental Science and Engineering, 71127 Nanjing University of Information Science and Technology, Nanjing 210044, China; ‡ School of Energy and Environment, 53025 City University of Hong Kong, Hong Kong 999077, China; § College of Environment and Climate, Institute of Mass Spectrometry and Atmospheric Environment, 47885 Jinan University, Guangzhou 510632, China; ∥ Department of Advanced Technologies in Medicine & Dentistry, University “G.d’Annunzio” of Chieti-Pescara, Chieti 66100, Italy; ⊥ Center for Advanced Studies and Technology (CAST), Chieti 66100, Italy; # Yangzhou Environmental Monitoring Center of Jiangsu Province, Yangzhou 225009, China; ∇ School of Energy and Environment, 12579 Southeast University, Nanjing 211189, China; ○ Yunnan Provincial Key Laboratory of Soil Carbon Sequestration and Pollution Control, Faculty of Environmental Science & Engineering, Kunming University of Science & Technology, Kunming 650500, China; ◆ School of Emergency Management, 71127 Nanjing University of Information Science and Technology; Nanjing 210044, China

**Keywords:** micro−nanoplastics (MNPs), bioaerosol single-particle mass spectrometry (Bio-SPAMS), polystyrene (PS), mixing state, real-time detection

## Abstract

Atmospheric micro–nanoplastics (MNPs) serve as key vectors for the global dispersion of plastic pollutants and act as reactive interfaces for atmospheric species, modifying their physicochemical properties and influencing environmental transport dynamics. However, existing methods lack the temporal resolution and specificity to characterize MNP mixing states and pollutant interactions in real time. To address this gap, we developed an innovative approach employing bioaerosol single-particle mass spectrometry (Bio-SPAMS) for simultaneous detection of polystyrene MNPs (PS MNPs; 0.3–2 μm) and their chemical associations with co-pollutants. Three diagnostic tracer ions, ^91^[C_7_H_7_^+^], ^104^[C_8_H_8_^+^], and ^115^[C_9_H_7_^+^], were identified as unambiguous markers of PS MNPs, enhancing their discrimination from ambient aerosols. Field measurements in a Chinese megacity revealed that PS MNPs constitute 1.04% of total aerosols (*n* = 51 045 particles), predominantly within the 0.3–0.8 μm size range. Approximately 76.4% of PS MNPs exhibited co-detection of nitrate and sulfate signatures, and in particles with PS characteristics, the relative peak areas of nitrate and sulfate are 14.30 and 4.06%, respectively, demonstrating active atmospheric aging via secondary pollutant uptake. This work established a new methodology for real-time MNP tracking in atmospheric matrices, providing critical insights into their lifecycle and risks.

## Graphical abstract

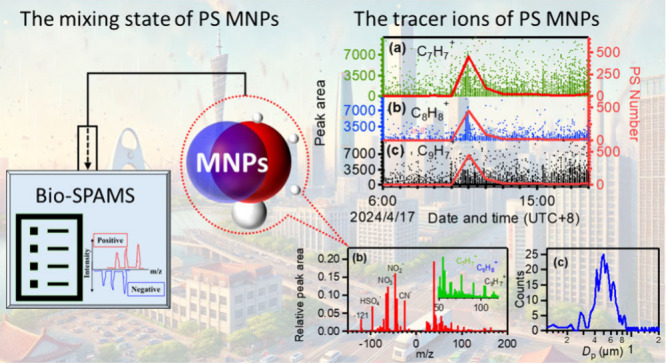

## 1 Introduction

Micro–nanoplastics (MNPs) have emerged as pervasive environmental contaminants,^1–3^ with atmospheric transport recognized as an important pathway for their global dispersion and human exposure.^4–8^ Beyond passive carriers, airborne MNPs dynamically interact with co-existing pollutants, forming complex mixtures that influence atmospheric processes, radiative forcing, and human health.^2,7,9–16^ The mixing state, defined as the molecular-scale association of MNPs with adsorbed species, critically governs their reactivity, toxicity,^17^ and climatic effects.^18^ For instance, microplastics have been shown to enhance the toxicity of florfenicol, an antimicrobial agent, by acting as carriers or disrupting metabolic processes.^19–22^ The ice-nucleating efficiency of MNPs can also be altered upon mixing with inorganics such as sulfuric acid and ammonium sulfate,^23^ which has implications for cloud formation and climate feedback. Despite this significance, real-time characterization of MNP mixing states at the single-particle level remains challenging, limiting mechanistic understanding of their atmospheric transformation and environmental effects.

Traditional methods for atmospheric MNPs analysis rely on offline techniques with inherent limitations.^24,25^ Visual identification, applicable only to millimeter-scale (1–5 mm) plastics, lacks the ability to identify polymer types or distinguish plastics from organics, and introduces observer bias.^26^ Fourier transform infrared (FTIR) and Raman spectroscopies resolve polymer types but suffer from diffraction limits (>10 μm resolution), environmental interference, and throughput constraints.^7,27–30^ Bulk mass spectrometry (MS) methods including pyrolysis–gas chromatography–mass spectrometry (Pyr–GC–MS), thermal desorption–gas chromatography–mass spectrometry (TDS–GC–MS), and matrix-assisted laser desorption/ionization time-of-flight mass spectrometry (MALDI–TOF MS), provide chemical specificity but require destructive sample processing, altering native particle morphologies and masking real-time interaction dynamics.^7,26,31–34^ While Aerodyne high-resolution time-of-flight aerosol mass spectrometry (HR-ToF-AMS) enables real-time detection of sub-micrometer polystyrene (PS) and polyethylene terephthalate (PET) particles (0.05–1.0 μm),^35,36^ its design fundamentally restricts mixing state analysis: aerodynamic focusing and high-temperature vaporization (600 °C) at the tungsten surface homogenize particle components and ionized by electron ionization (70 eV), precluding single-particle speciation.^37–40^ In contrast, single-particle aerosol mass spectrometry (SPAMS) overcomes these limitations by coupling aerodynamic sizing with dual-polarity time-of-flight detection.^40,41^ Using laser desorption/ionization, it vaporizes the entire particle and detects core and associated species via time-of-flight mass spectrometry. This enables direct identification of internally mixed components, distinguishing particles with complex chemical associations from externally mixed ones.^42–46^

This study pioneers the application of Bio-SPAMS for *in situ* chemical characterization of polystyrene micro–nanoplastics (PS MNPs) and their atmospheric mixing states. We analyzed both laboratory-generated PS MNPs and ambient aerosols sampled in urban Guangzhou, China. Co-detection of sulfate and nitrate on most of PS MNPs reveals rapid atmospheric aging, forming chemically complex aerosols that may modulate atmospheric reactivity, particle nucleation processes, and toxicity profiles. These findings would provide critical parametrizations for atmospheric models, specifically, MNP number concentrations, size-resolved aging rates, and surface uptake coefficients, enabling robust assessment of their climate and health impacts.

## 2 Experimental Methods

The Bio-SPAMS (Nanjing Feng-Sun Intelligent Technology Co., Ltd., China) were employed in all the laboratory experiments and ambient sampling of this study. The Bio-SPAMS is developed based on the SPAMS^47,48^ and is primarily composed of control and acquisition software, a sampling system, a diameter measurement system, an ionization system, and a mass spectrometry system to characterize aerosol particle size and chemical composition at the single-particle level.

The instrument employs aerodynamic lenses to focus aerosol particles into a particle beam for introduction into a vacuum chamber. The focused particle beam subsequently traverses two continuous-wave (CW) diode-pumped neodymium-doped yttrium aluminum garnet (Nd:YAG) laser beams (λ = 532 nm) for real-time aerodynamic sizing of individual particles via light scattering. Following sizing, particles are precisely positioned within the ionization source chamber, where a pulsed Nd:YAG laser (λ = 266 nm) generates simultaneous positive and negative ion fragments through photoionization and dissociative electron processes. The ionized products are then detected using a dual-polarity time-of-flight mass spectrometer (dual TOF-MS), enabling the measurement of distinct chemical components within individual aerosol particles. The system’s design principles are detailed in the literature by Du et al.^47^ and Li et al.^48^

Prior to sampling, the Bio-SPAMS requires particle size calibration and mass spectrometry calibration, with detailed calibration procedures provided in Text S1, and the sampling flow rate was set at 0.35 L min^–1^, while the ionization laser energy was adjusted under exploratory conditions, as detailed in section 3.2. The chemical composition and particle sizes of single particles collected by the SPAMS were analyzed using MATLAB and the Version 1.3 of Computational Continuation Core (COCO V1.3).^46^ In this study, the programs of “averaged and digitized spectra”, “particle size distribution”, and “search for particles with specific conditions” were mainly used for data analysis.

The HR-TOF-AMS (Aerodyne Research, Inc., U.S.A.) was conducted parallelly to measure the total concentration of PS NNPs, as reported by Niu et al.^35^ The working principle of the HR-TOF-AMS is extensively detailed in the studies by Decarlo et al.^49^ and Jayne et al.^38^ Ionization efficiency (IE) and particle size calibration were performed in accordance with the protocols outlined by Canagaratna et al.^50^ and Drewnick et al.^40^ In this study, particles were sampled at a flow rate of 1.43 cm^3^ s^–1^, vaporized at approximately 600 °C, with the mass spectrometer operating in V-mode.^51^ The HR-ToF-AMS data set was analyzed by using the Igor-based standard ToF-AMS Analysis Toolkit SOUIRREL v1.59D and PIKAv1.19D (https://cires1.colorado.edu/jimenez-group/wiki/index.php?title=ToF-AMS_Main).

To acquire the standard mass spectrum of pure PS MNP, an aqueous solution of 500 nm monodispersed PS particles (Thermo Scientific, 8 wt %) was generated by a constant-output atomizer (TSI, Inc., model 9302), and concurrently analyzed by both Bio-SPAMS and HR-ToF-AMS. The sampling was stopped after 5000 mass spectra were acquired by the Bio-SPAMS. The experimental setup is depicted in Figure 1a.

**Figure 1:**
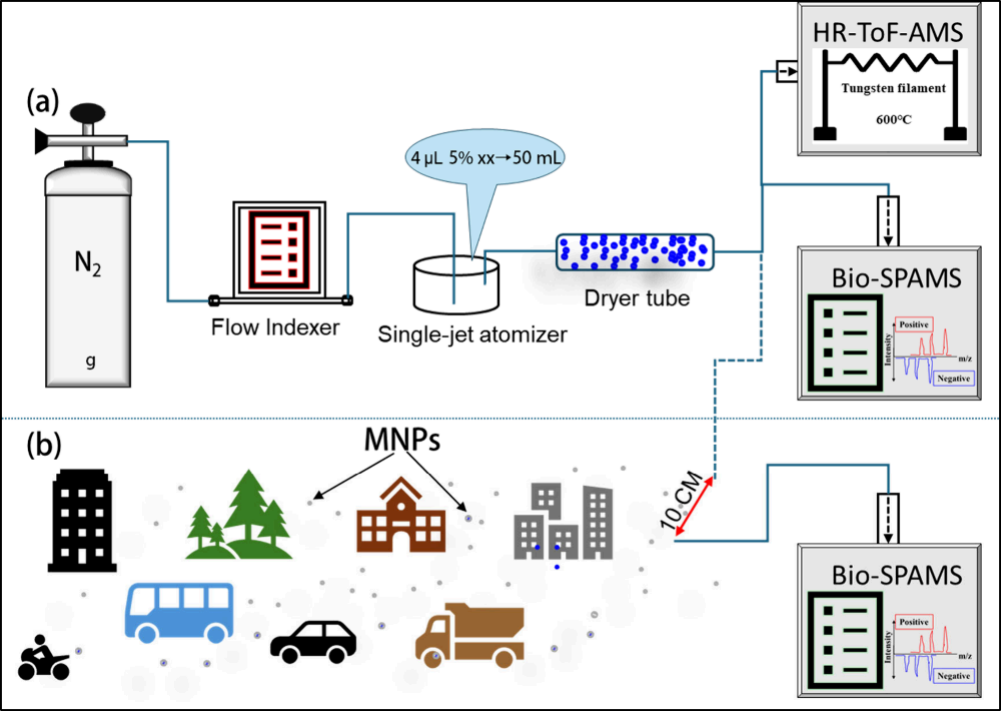
Schematic of the experimental setup: (a) experimental setup for laboratory aerosol generation and detection and (b) experimental setup for ambient aerosol detection with standard PS (“10 CM” in panel b refers to the distance from the spray nozzle to the sampling inlet).

The laser energy and particle size were further tested to evaluate the influence of different conditions on PS MNPs using Bio-SPAMS. The optimization of laser energy was investigated by testing six discrete energy levels (300–1550 μJ; Table S1) at a constant particle size of 500 nm. Particle size-dependent collection efficiency was evaluated by analyzing PS MNPs (Thermo Scientific, 8 wt %) ranging from 0.3 to 2 μm within the optimized laser energy range, at ∼380 μJ based on prior optimization. The specific experiment involved aerosolizing an aqueous solution containing pure monodisperse PS particles using the atomizer, followed by collection and analysis with Bio-SPAMS (Figure 1a).

To assess the capability of detecting mixed states, a mixed solution was aerosolized using the atomizer and collected by Bio-SPAMS within the optimized laser energy range. The mixed solution was prepared by combining 500 nm PS MNPs with equal volumes of ammonium sulfate (AS, 8 wt %, 4 μL) and sodium nitrate (SN, 8 wt %, 4 μL) in ultrapure water. To distinguish PS MNP-specific signals from potential interferents, comparative analyses were conducted using literature-derived data, and incense combustion was detected by Bio-SPAMS within the optimized laser energy range. Detailed findings are presented in section 3.3. The experimental processes are detailed in Text S2.

Ambient aerosol samples were collected over a 3 day period in Huangpu District, Guangzhou, a megacity characterized by a large permanent population and intensive plastic consumption, to investigate particulate matter composition under high anthropogenic influence. During sampling, the 500 nm PS through the experimental procedures of Figure 1a was sprayed into the air 3 times (under normal ventilation conditions), each time for 5 min, and the spray experiments were conducted in a well-ventilated outdoor, ventilated environment. This spray port is 10 cm from the sampling port (Figure 1b), and the sampling port is about 9 m above the ground.

## 3 Results and Discussion

### 3.1 Mass Spectrum of Pure PS MNP Standard

The mass spectral signatures of pure PS MNPs obtained via Bio-SPAMS were compared with parallel measurements using HR-ToF-AMS to evaluate detection capabilities (Figure 2). The mass spectrum of pure PS MNPs using Bio-SPAMS at 384.24 ± 5.68 μJ revealed a fragmentation profile dominated by hydrocarbon ions (^*n*^[C_*x*_H_*y*_^±^]) (Figure 2a), including *m*/*z* ^25^[C_2_H^–^], ^49^[C_4_H_5_^–^], ^39^[C_3_H_3_^+^], ^51^[C_4_H_3_^+^], ^63^[C_5_H_5_^+^], ^77^[C_6_H_5_^+^], ^91^[C_7_H_7_^+^], ^104^[C_8_H_8_^+^], and ^115^[C_9_H_7_^+^], which is mostly consistent with the aromatic–aliphatic backbone. Compared with Bio-SPAMS (Figure 2a), the results by HR-ToF-AMS align with the positive mass spectrum by Bio-SPAMS, which detected analogous ions in Figure 2b (*m*/*z* ^39^[C_3_H_3_^+^], ^51^[C_4_H_3_^+^], ^63^[C_5_H_5_^+^], ^77^[C_6_H_5_^+^], ^91^[C_7_H_7_^+^], ^104^[C_8_H_8_^+^], ^115^[C_9_H_7_^+^], and ^193^[C_15_H_13_^+^]), are consistent with previous results^35^ as well as the mass spectra of styrene in the NIST library, confirming PS MNPs identifiable spectral fingerprints across both methods. Bio-SPAMS is also able to capture aliphatic fragments in negative ion mode (*m*/*z* ^25^[C_2_H^–^] and ^49^[C_4_H_5_^–^]). The *m*/*z* 104 peak (^104^[C_8_H_8_^+^]), corresponding to styrene monomer release, was consistently observed in both methods but exhibited higher relative intensity in Bio-SPAMS. These findings demonstrate that Bio-SPAMS can detect PS MNPs through characteristic spectral fingerprints. However, the complete mass spectrometry of PS still needs to be further explored and, experimental conditions also need to be further optimized, since the detection results by Bio-SPAMS are affected by the laser energy, the particle size, and the composition.^47,52,53^

**Figure 2:**
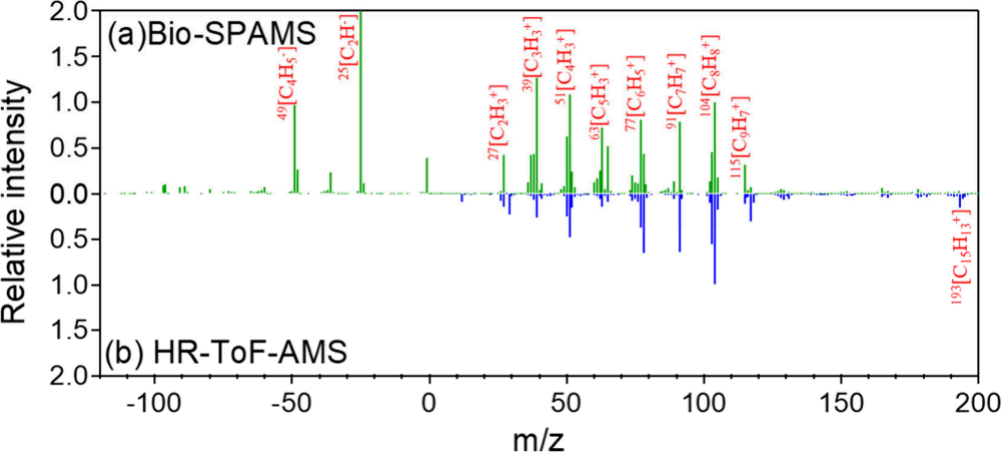
Mass spectra of PS MNPs detected by Bio-SPAMS and HR-TOF-AMS: (a) mass spectra of pure PS by Bio-SPAMS and (b) mass spectra of pure PS by HR-ToF-AMS (relative intensities obtained in terms of peak area or concentration of C_8_H_8_^+^).

### 3.2 Optimized Detection Conditions

To optimize the detection conditions for PS MNPs using Bio-SPAMS, we further investigated the effects of laser energy and particle size on detection efficiency and mass spectral completeness (Figure 3 and Figures S1 and S2).

**Figure 3:**
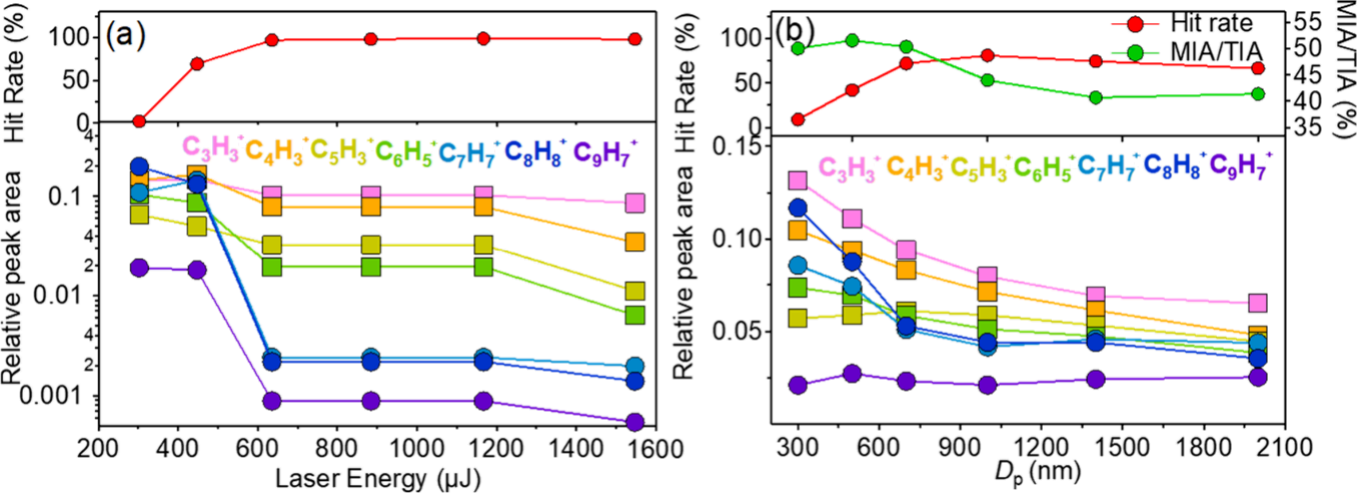
PS was detected by Bio-SPAMS: (a) detection efficiency of 500 nm PS MNPs at different laser energies and (b) detection efficiency of PS MNPs at different sizes (MIA, major ion abundance stands for total relative peak areas of major ions; TIA, total ion abundance stands for total relative peak area of total ions; MIA/TIA, major ion abundance to total ion abundance ratio; and MIA/TIA enables quantitative assessment of the proportion of major target ions within total ionization products).

#### 3.2.1 Impact of Laser Energy on Detection Performance

Laser energy is a key parameter influencing both the ionization efficiency and the fragmentation pattern of aerosol particles. Thus, we conducted experiments detecting 500 nm PS MNPs at six laser energy levels (300–1550 μJ) (Table S1) by Bio-SPAMS, collecting 5000 mass spectra at each setting. The average mass spectra for each laser energy is presented in Figure S1 of the Supporting Information. The results revealed that increasing laser energy leads to a reduction in the signal intensity of large fragment ions in positive ion mode, e.g., ^91^[C_7_H_7_^+^], ^104^[C_8_H_8_^+^], and ^115^[C_9_H_7_^+^] (Figure 3a and Figure S1), while in negative ion mode, although the variety of fragment ions increases, the signal strength of key ions, e.g., ^25^[C_2_H^–^] and ^49^[C_4_H_5_^–^] diminishes (Figure S1). This observation aligns with previous studies Silva and Prather,^54^ which reported that higher laser energies promote further fragmentation of large ions into smaller species. Thus, lower laser energies are more effective for preserving large fragment ions, which are critical for identifying target compounds.^55,56^

Since there is no standard mass spectral library for laser ionization resolution, but according to the existing research results, molecular ions generally occur at low laser energies.^47,54^ Therefore, it can be known a more complete mass spectrum of PS MNPs can be obtained in the range of laser energies from 301.87 ± 6.12 to 635.61 ± 33.11 μJ, because the large ion fragment C_9_H_7_^+^ has remained present under the range of laser energy (301.87 ± 6.12 to 635.61 ± 33.11 μJ) (Figure 3a and Figure S1). Although a more complete mass spectrum of PS MNPs can be obtained in the range of laser energies from 301.87 ± 6.12 to 635.61 ± 33.11 μJ, the detection efficiency (including hit rate) at different laser energies still needs to be considered in the experiment.^47^

The hit rate reaches the stable highest level of about 96.89–98.72% between 635.61 ± 33.11 and 1200 μJ (Figure 3a). However, when laser energy exceeds 635.61 ± 33.11 μJ, the signals for critical ions such as C_7_H_7_^+^, C_8_H_8_^+^, and C_9_H_7_^+^ (Figure 3a) nearly disappeared. Previous studies have demonstrated that excessive laser energy can lead to non-specific fragmentation and reduced ionization efficiency for specific compounds.^47,54^ To balance the retention of key fragment ions with a high hit rate, the recommended laser energy range should be maintained above 301.87 ± 6.12 μJ and kept below 635.61 ± 33.11 μJ for optimal performance. Li et al.^48^ have reported that a pulsed 266 nm Nd:YAG laser (Ultra Quantel, France) with adjustable energy from 0.1 to 10 mJ is commonly used for particle ionization, a energy range of 301.87 to 635.61 μJ falls well within this operational window. This range ensures sufficient ionization while minimizing excessive fragmentation, there by preserving the characteristic spectral fingerprints of PS MNPs.

#### 3.2.2 Impact of Particle Size on Detection Efficiency

Particle size is another critical factor influencing the performance.^47,57^ Thus, we detected PS MNPs of six sizes (300 nm, 500 nm, 700 nm, 1 μm, 1.4 μm, and 2 μm) under the same optimized laser energy range (above 301.87 ± 6.12 μJ and below 635.61 ± 33.11 μJ) by Bio-SPAMS. The results confirm that PS MNPs across the 0.3 to 2 μm size range can be effectively detected by Bio-SPAMS, with consistent identification of key fragment ions (e.g., C_2_H^–^, C_4_H_5_^–^, C_3_H_3_^+^, C_4_H_3_^+^, C_5_H_5_^+^, C_6_H_5_^+^, C_7_H_7_^+^, C_8_H_8_^+^, and C_9_H_7_^+^), as observed in Figures 2a and 3a.

However, the relative peak areas of key fragment ions varied across different particle sizes (Figure S2 and Figure 3b). For instance, 500 and 700 nm PS MNPs exhibited significantly higher relative peak areas for C_2_H^–^, while 300 and 500 nm PS MNPs showed greater relative peak areas for C_8_H_8_^+^. Moreover, we also calculated the ratio of the total relative peak area of major ions to that of all ions (MIA/TIA) to evaluate detection efficiency comprehensively. The 500 nm PS MNPs demonstrated the highest MIA/TIA ratio (51.54%), indicating superior detection efficiency for key fragment ions. Additionally, 500 nm PS MNPs achieved the highest hit rate of 80.88% (Figure 3b). These results suggest that 500 nm PS MNPs are optimal for experimental detection, as they provide a balance between signal intensity and detection efficiency.

Therefore, the optimal conditions for detecting PS MNPs with Bio-SPAMS are achieved by adjusting the laser energy to between 301.87 ± 6.12 and 635.61 ± 33.11 μJ, which balances the preservation of key fragment ions with a high hit rate. Additionally, particle sizes between 500 and 700 nm maximize detection efficiency and ensure high spectral integrity. The mass spectra obtained from Bio-SPAMS under the aforementioned optimal conditions (Figure 2a) were selected as the standard spectra (fingerprint) for subsequent studies.

### 3.3 Identification of PS MNP Tracer Ions

To accurately extract PS MNPs from complex environmental samples, it is essential to identify unique tracer ions that can distinguish PS MNPs from interfering species. Previous studies have highlighted the challenges of detecting MNPs in atmospheric matrices due to potential interferences from structural similarities in organic components^58^ such as organic carbon (OC),^44,57^ biomass burning (BB),^44,57–60^ and polycyclic aromatic hydrocarbons (PAHs).^61,62^ These substances can produce ions similar to PS MNPs, complicating the detection process.

Thus, we further analyzed the mass spectra of PS MNPs and potential interferents to identify characteristic tracer ions. As shown in Table S2, common interfering ions, e.g., ^25^[C_2_H^–^], ^49^[C_4_H_5_^–^], ^39^[K^+^], ^51^[C_4_H_3_^+^], ^63^[C_5_H_5_^+^], and ^77^[C_6_H_5_^+^] primarily originate from OC, BB, and PAHs. To exclude these interferences, we focused on ions that are unique to PS MNPs. Our results demonstrated that ^91^[C_7_H_7_^+^], ^104^[C_8_H_8_^+^] and ^115^[C_9_H_7_^+^] do not appear simultaneously in the mass spectra of burning incense (Figure 4) (biomass burning organic aerosol, BB) and standard PAHs^62^ at 500 μJ by SPAMS. Thus, C_7_H_7_^+^, C_8_H_8_^+^ and C_9_H_7_^+^ can be considered as tracer ions for PS MNPs from laser ionization at an energy between 301.87 ± 6.12 and 635.61 ± 33.11 μJ by Bio-SPAMS.

**Figure 4:**
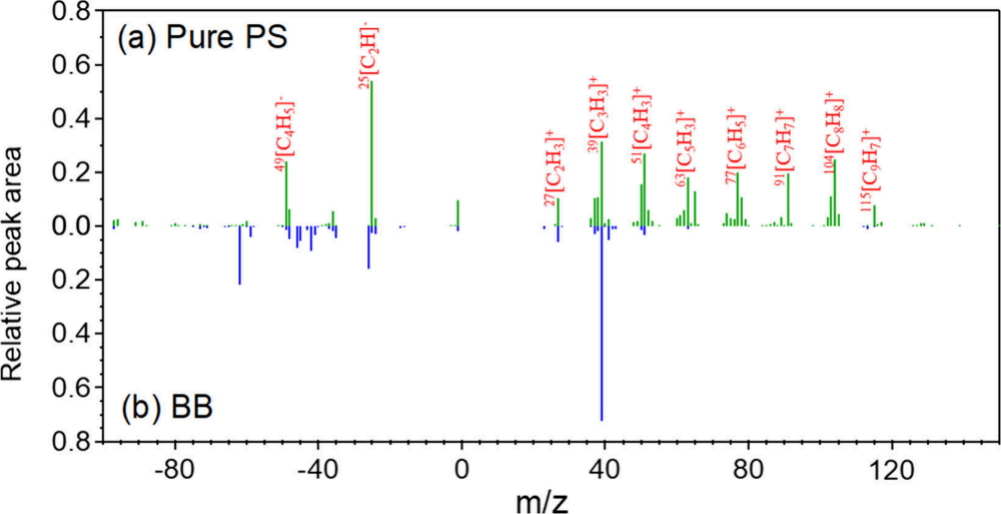
Mass spectra of pure PS and incense burning particles: (a) mass spectra of pure 500 nm PS under optimized energy conditions and (b) mass spectra of the burning incense under optimized energy conditions.

### 3.4 Identification of Mixing States of PS MNPs with Other Components

We conducted experiments by mixing standard 500 nm PS MNPs with common inorganic substances, specifically ammonium sulfate (AS) and sodium nitrate (SN). An average mass spectrum of 5000 single particles from a mixture of PS, AS, and SN, collected by the Bio-SPAMS is shown in Figure 5. Compared with the mass spectra of pure PS MNPs (Figure 5a), the mass spectra of the mixture exhibit clear signals for nitrate and sulfate ions (Figure 5a), the relative intensity of nitrate (*m*/*z* ^62^[NO_3_^–^], ^46^[NO_2_^–^], and ^30^[NO^–^]) is about 0.94 and that of sulfate­(*m*/*z* ^97^[HSO_4_^–^], ^96^[SO_4_^–^], ^81^[HSO_3_^–^], and ^80^[SO_3_^–^]) is about 1.46 in mass spectra of mixture, while in mass spectra of pure, the relative intensity of nitrate is about 0.027 and that of sulfate is about 0.23. Furthermore, the particle size distribution of the mixture showed a broader range, with most particles exceeding 500 nm (Figure 5b). This increase in particle size suggests that PS MNPs may be in a state of embedment or surface adsorption with sulfate and nitrate.^15^ These results indicate that our method can identify the mixing state of MNPs with the co-existing species at the individual particle level.

**Figure 5:**
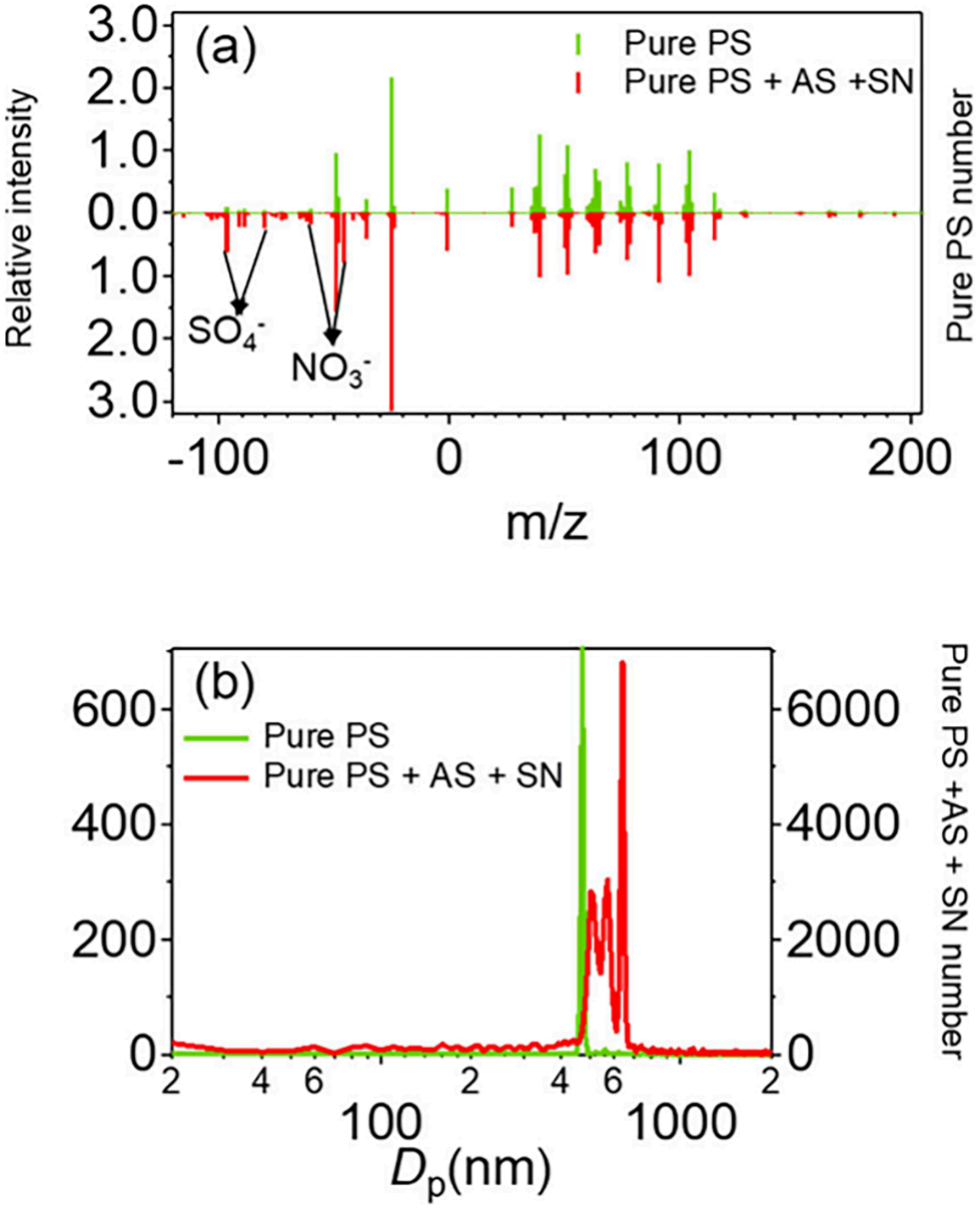
PS internally mixed with common inorganics: (a) mass spectra of pure 500 nm PS, (b) mass spectra of mixture (sodium nitrate, ammonium sulfate, and PS MNPs), and (c) particle size distribution of mixture (sodium nitrate, ammonium sulfate, and PS MNPs) (relative intensity obtained in terms of peak area of C_8_H_8_^+^).

### 3.5 Validation and Application to the Detection of Atmospheric PS

Atmospheric aerosol samples with labeled PS MNPs (500 nm) were collected by Bio-SPAMS under optimized conditions (laser energy: 410.85 ± 6.06 μJ) from April 15 to 18, 2024. A total of 122 348 particles with mass spectra were collected. Using ^91^[C_7_H_7_^+^], ^104^[C_8_H_8_^+^], and ^115^[C_9_H_7_^+^] as qualitative fragment ions, we applied a relative peak area threshold of ≥0.005 for ^104^[C_8_H_8_^+^] based on Zhang et al.^62^ and ion signal intensity. This threshold, well above baseline levels, minimized background noise interference and ensured reliable detection of low-abundance MNPs. Under these criteria, 3198 particles exhibiting PS MNP characteristics were identified, representing 2.61% of total particles. Time-series analysis revealed a clear correlation between the release of standard PS MNPs into the atmosphere and the detected number of PS MNP-like particles (Figure 6a–c). During the periods when labeled PS MNPs were introduced, the peak areas of ^91^[C_7_H_7_^+^], ^104^[C_8_H_8_^+^], and ^115^[C_9_H_7_^+^] showed a significant increase, especially, the changes in the peak area of ^104^[C_8_H_8_^+^] are more synchronized with the changes in the quantity of labeled PS MNPs, confirming the reliability of these tracer ions in extracting PS MNPs from complex environmental matrices.

**Figure 6:**
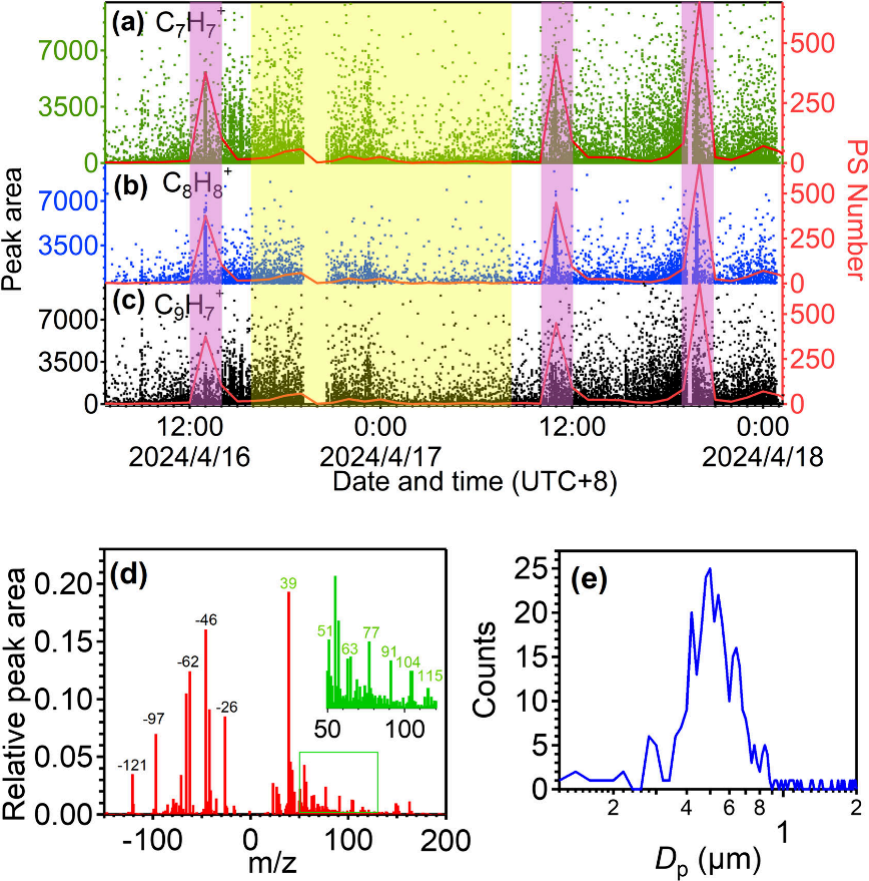
Ambient measurement of PS with Bio-SPAMS in an urban environment in Guangzhou: (a, b, and c) peak area of ^91^[C_7_H_7_^+^], ^104^[C_8_H_8_^+^], and ^115^[C_9_H_7_^+^] in the ambient aerosol and the change of PS MNPs searched (each purple-shaded area is the time period when the standard PS was sprayed into the atmosphere for 5 min, and the yellow-shaded region is the time periods of the ambient aerosol), (d) mass spectra of particles characterized as PS were detected in the yellow-shaded region, and (e) particle size distribution of particles characterized as PS were detected in the yellow-shaded region.

A total number of 51 045 ambient aerosols without labeled PS MNPs collected for 27.5 h by Bio-SPAMS (yellow-shaded regions in Figure 6a, b, and c) demonstrated that PS MNPs accounted for approximately 1.04% of the total detected particles (Figure 6d and e). Notably, 76.42% of these PS MNPs exhibited strong signals for nitrate and sulfate (e.g., *m*/*z* ^97^[HSO_4_^–^], ^96^[SO_4_^–^], ^81^[HSO_3_^–^], and ^80^[SO_3_^–^] and *m*/*z* ^62^[NO_3_^–^], ^46^[NO_2_^–^], and ^30^[NO^–^]), and in particles with PS characteristics, the relative peak area of nitrate and sulfate are 14.30 and 4.06%, respectively (Figure 6d), these findings suggest that PS MNPs may interact with co-existing inorganic pollutants. The particle size is mainly concentrated in the range of 0.3–0.8 μm (Figure 6e). Thus, microplastics may act as carriers for other pollutants, enhancing their environmental persistence and transportability.^15^

The results showed that the content of PS MNPs about 0.3–0.8 μm was significantly lower than that of conventional pollutants in the atmosphere in Guangzhou. Similarly, the results of Niu et al. also showed that the mass concentration of PS NPS in the atmosphere of Texas was 30 ± 20 ng m^–3^ by HR-TOF-AMS, which was lower than that of conventional pollutants.^35^ Kirchsteiger et al.^63^ reported that in Graz, Austria, the microplastic content was 0.67% of PM_2.5_ by thermal desorption proton transfer reaction mass spectrometry (TD–PTR–MS). This indicates that the current atmospheric content of MNPs remains at an extremely low level compared to conventional pollutants such as sulfates, nitrates, black carbon, and mineral dust.

However, the low concentration of MNPs does not equate to negligible environmental impacts. Due to their strong environmental persistence and global cycling capacity, MNPs may accumulate invisibly, and their potential risks could manifest through complex interactions with other pollutants in the atmosphere.^1^ This suggests that some other air contaminants can interfere with the detection of PS MNPs (e.g., masking the signal from PS MNPs, wrapping around the surface of PS MNPs, etc.). Besides, the particles with PS characteristics, also have extremely strong nitrate and sulfate signals, which may be due to their surface properties (e.g., charge distribution and functional group formation), environmental conditions (e.g., humidity), and interaction with other particulate matter.^64^

## 4 Atmospheric Implications

This study achieved real-time, single-particle identification of PS MNPs in urban aerosols through diagnostic tracer ions: ^91^[C_7_H_7_^+^], ^104^[C_8_H_8_^+^], and ^115^[C_9_H_7_^+^], which provide unambiguous differentiation from organic/inorganic interferents using Bio-SPAMS. Systematic optimization of desorption/ionization parameters revealed an operational laser energy window of 301.87 ± 6.12 to 635.61 ± 33.11 μJ. Within this range, PS MNPs (500–700 nm) exhibited maximal detection efficiency and reproducible fragmentation patterns. Field measurements in urban Guangzhou quantified PS MNPs at 1.04% of ambient aerosols, most of them were in the 0.3–0.8 μm size range. These findings highlight the predominance of respirable nanoplastics and underscore the necessity for sub-micrometer analytical resolution. In addition, approximately 76.4% of PS MNPs co-detected nitrate and sulfate enrichments, suggesting dynamic interactions with co-existing pollutants during atmospheric processing. This frequent co-detection suggests that PS MNPs undergo atmospheric aging via condensation or heterogeneous reactions with secondary inorganic aerosols. Such transformations likely enhance particle hygroscopicity, atmospheric lifetime, and deposition behavior, potentially increasing their ability to act as ice nucleating particles (INPs) or to penetrate deeper into the human respiratory tract.^19–23,65^

Importantly, the mixing state and particle size information derived from tracer ions offers a valuable foundation for the parametrization of PS MNP aging in atmospheric models. For instance, reaction rates or coating efficiencies could be constrained using observed nitrate/sulfate associations, analogous to existing schemes for soot or organic aerosol aging.^66,67^ Incorporating such dynamics would improve model predictions of plastic particle transport, transformation, and deposition under variable environmental conditions.^66–68^ Overall, this study bridges analytical innovation with environmental relevance by unveiling not only the presence of airborne PS MNPs in urban environments but also their chemical evolution during atmospheric processing. These insights contribute toward a mechanistic understanding of nanoplastic–pollutant interactions and facilitate their integration into multiscale models that address aerosol–climate–health linkages. In the context of global change, the dynamic behavior of airborne MNPs deserves increased attention as both a novel pollutant and an active participant in the atmospheric system.

However, one limitation of this study is the potential underrepresentation of particles smaller than 300 nm and larger than 1 μm. The former is challenging to detect due to their low mass, which results in weak ion signals and inefficient laser ionization, while the latter may be lost because of aerodynamic lens transmission inefficiencies^69^ and sampling losses. These size ranges are environmentally relevant: sub-300 nm particles tend to be more bioavailable, whereas coarse particles (>1 μm) can carry higher loads of sorbed pollutants. To address this gap, future research should focus on enhancing the sensitivity and transmission efficiency of single-particle mass spectrometry platforms, enabling more comprehensive detection and characterization across the full size spectrum of atmospheric MNPs.

## Supplementary Material



## Notes

Instrument description and calibration of Bio-SPAMS (Text S1), preparation of laboratory-generated aerosol samples and cleaning protocols (Text S2), mass spectra of PS at different laser energies (Figure S1), mass spectra of PS at different particle sizes (Figure S2), six different laser energies (Table S1), and ion markers for different particle types measured by SPAMS (Table S2) (PDF)
