# Supplementary material for: Real-Time Detection of Urban Atmospheric Micro–Nanoplastics and Their Chemical Mixing State Using Bioaerosol Single-Particle Mass Spectrometry
Source: Environ Sci Technol. 2025 Sep 29;59(40):21600–8. doi: 10.1021/acs.est.5c06513 (PMC12529945; doi:10.1021/acs.est.5c06513)
Supplement: Supplementary file 1 [file es5c06513_si_001.pdf]

Supplemental Materials for

Real-time detection of urban atmospheric micro-nanoplastics and their chemical mixing state using bioaerosol single particle mass spectrometry

Chongchong Zhang, Yiming Qin*, Lei Li, Eleonora Aruffo, Shaoyong Li, Xuan Li, Ning Zhang, Yun Wu, Haiwei Li, Yunjiang Zhang, Yuan Dai, Ming Wang, Xinlei Ge, Ke Li, Wei Du, Chunlei Cheng, Mei Li, Mindong Chen, Junfeng Wang*

(*) Correspondence to:

Yiming Qin (ymqin3@cityu.edu.hk), ORCID: 0000-0002-1552-5139

Junfeng Wang (wangjunfeng@nuist.edu.cn), ORCID: 0000-0001-6215-1953

The supplemental materials have 7 pages, 2 supporting texts, 2 figures, and 2 tables:

Text S1. Instrument description and calibration of Bio-SPAMS

Text S2. Preparation of laboratory-generated aerosol samples and cleaning protocols

Figure S1. Mass spectra of PS at different laser energies

Figure S2. Mass spectra of PS at different particle sizes

Table S1. Six different laser energies

Table S2. Ion markers for different particle types measured by SPAMS

Text S1. Instrument description and calibration of Bio-SPAMS:

The detailed principle and structure of Bio-SPAMS have been previously reported by Du et al. (2024)[1] and Li et al. (2011)[2]; thus, only the principle of the instrument is briefly stated here. Bio-SPAMS is primarily composed of control and acquisition software, a sampling system, a diameter measurement system, an ionization system, and a mass spectrometry system. The instrument adopts a 266-nm Nd: YAG ultraviolet pulse laser, and the logic control is realized by a time sequence circuit. By accurately calculating the drift speed of the particles, the time sequence circuit generates a laser emission signal when the particles reach the center of the ionization zone, and the emitted pulse laser ionizes the particles. Now, Bio-SPAMS can detect particle sizes ranging from 150 to 5000 nm[1].

The calibration of Bio-SPAMS is bifurcated into two distinct components: particle size calibration and mass spectrometry calibration. The particle size calibration is achieved by employing an aerosol generator to produce polystyrene latex spheres (PSLs) of standardized diameters (0.2, 0.3, 0.5, 0.72, 1.0, 1.3, and 2.0 μm), which serve to correct the particle size measurements and ensure the precision of the detected particle diameters. To guarantee the accuracy of mass spectrometry detection, To ensure the accuracy of mass spectrometry detection, mass spectrometry drift calibration was performed using the primary substance fragments $^{23}[Na^+]$, $^{39}[K^+]$, $^{208}[Pb^+]$, $^{46}[NO_2^-]$, $^{62}[NO_3^-]$ and $^{97}[HSO_4^-]$. It is noted that the calibration of this instrument was conducted by engineers prior to the commencement of the experiments in this study.

Text S2. Preparation of laboratory-generated aerosol samples and cleaning protocols:

1) Preparation of laboratory-generated aerosol samples: All stock solutions were initially prepared at a mass concentration of 8%. For pure compound experiments, 4 μL of a single solution was diluted with ultrapure water to a final volume of 50 mL. For mixture experiments, 4 μL of each solution was combined in equal volumes and similarly diluted to 50 mL with ultrapure water. The prepared solution was sonicated for 10 minutes to ensure thorough mixing, and then transferred into a clean single-jet atomizer. The atomizer was subsequently connected to a new drying tube and a nitrogen

gas line (flow rate: 2 L/min). The generated aerosol was then introduced into the sampling inlet of the Bio-SPAMS or HR-ToF-AMS for online analysis.

2) cleaning protocols: During the experimental procedure, all glassware utilized was cleaned three times with ultrapure water and ethanol. After measuring each sample, we will clean the inlet orifice with anhydrous ethanol to ensure that the inlet is unobstructed and the pressure is normal, then the aerosol generator was sonicated for approximately 30 min and it was cleaned with ultrapure water three times and then wiped clean with dust-free paper to eliminate the background interference. Subsequently, ultrapure water in the aerosol generator was introduced into the entire device until no new single particle spectrum was generated, and then the next sample could be measured.

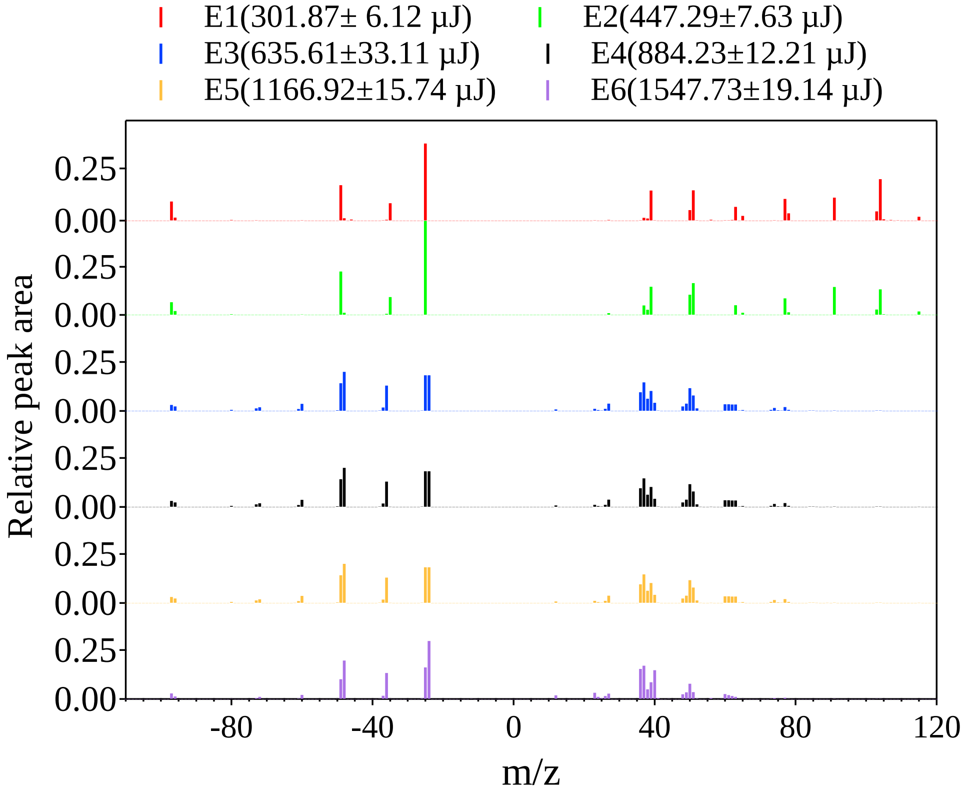

Figure S1. Mass spectra of PS at different laser energies

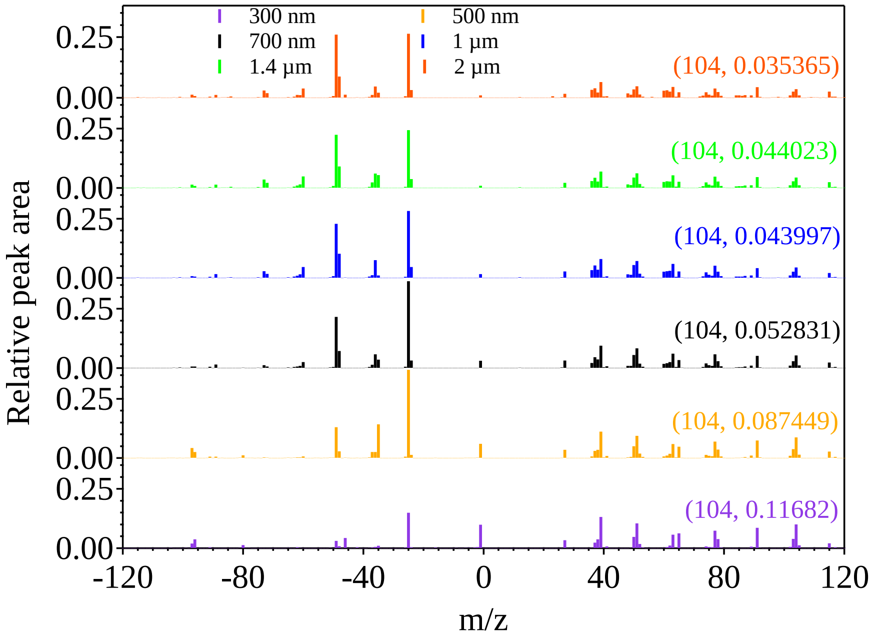

Figure S2. Mass spectra of PS at different particle sizes

Table S1 Six different laser energies

class	E1	E2	E3	E4	E5	E6
Energy（μJ）	301.87	447.29	635.61	884.23	1166.92	1547.73
SD	±6.12	±7.63	±33.11	±12.21	±15.74	±19.14

Table S2 Ion markers for different particle types measured by SPAMS

Type	Positive ions	Negative ions	Reference
EC	EC: $^{12n}[C_n^+]$	EC: $^{12n}[C_n]^-$; sulfate: $^{80}[SO_3^-]$, $^{96}[SO_4^-]$, $^{97}[HSO_4^-]$,	3, 4-6
OC	$^{27}[C_2H_3]^+$, $^{37}[C_3H^+]$, $^{38}[C_3H_2^+]$, $^{43}[C_2H_3O^+]$, $^{51}[C_4H_3^+]$, $^{61}[C_5H^+]$, $^{63}[C_5H_3^+]$, $^{73}[C_6H^+]$, $^{74}[C_6H_2^+]$, $^{77}[C_6H_5^+]$, and $^{87}[C_7H_3^+]$, $^{17}[NH_3^+]$, $^{18}[NH_4^+]$, $^{30}[NO^+]$	EC; sulfate; nitrate:$^{62}[NO_3^-]$, $^{46}[NO_2^-]$; $^{26}[CN^-]$	3-5
BB	$^{39}[K^+]$, aromatic species ($^{51}[C_4H_3^+]$ and $^{63}[C_5H_3^+]$)	levoglucosan ($^{45}[CHO_2^-]$, $^{59}[C_2H_3O_2^-]$, $^{73}[C_3HO_3^-]$), $^{26}[CN^-]$, $^{35/37}[Cl^-]$, $^{42}[CNO^-]$	3, 5, 7
PAHs	$^{128}[C_{10}H_8^+]$, $^{152}[C_{12}H_8^+]$, $^{178}[C_{14}H_{10}^+]$, $^{179}[C_{14}H_{11}^+]$, $^{202}[C_{16}H_{10}^+]$, $^{203}[C_{16}H_{11}^+]$, $^{227}[C_{18}H_{11}^+]$, $^{228}[C_{18}H_{12}^+]$, and $^{235}[C_{19}H_7^+]$	$^{16}[O^-]$, $^{24}[C_2^-]$, $^{25}C_2H^-]$, $^{36}[C_3^-]$, $^{46}[NO_2^-]$, $^{48}[C_4^-]$, $^{49}[C_4H_5^-]$, $^{60}[C_5^-]$, $^{72}[C_6^-]$	8-11
K-rich	$^{39/41}[K+]$, EC, OC, amine	sulfate; nitrate;$^{35/37}[Cl^-]$; organonitrogen: $^{26}[CN^-]$, $^{42}[CNO^-]$	3, 4, 6
Ca-rich	$^{40}[Ca^+]$; $^{56}[CaO^+]$; $^{57}[CaOH^+]$; $^{75}[CaCl^+]$; $^{96}[Ca2O^+]$	sulfate; nitrate; $^{35/37}[Cl^-]$; $^{26}[CN^-]$; EC; phosphate: $^{63}[PO_2^-]$; $^{79}[PO_3^-]$; $^{95}[PO_4^-]$	3, 6
Fe-rich	$^{54/56/57}[Fe^+]$; $^{73}[FeOH^+]$	sulfate; nitrate; $^{35/37}[Cl^-]$; $^{26}[CN^-]$; EC; $^{16}[O^-]$; $^{17}[OH^-]$	3-5
Na-rich	$^{23}[Na^+]$; $^{39}[NaO^+]$; $^{54/56}[Fe^+]$; $^{46}[Na_2^+]$; $^{62}[Na_2O^+]$; $^{63}[Na_2OH^+]$; $^{81/83}[Na_2Cl^+]$	sulfate; nitrate; $^{35/37}[Cl^-]$; $^{26}[CN^-]$; EC; $^{16}[O^-]$; $^{17}OH^-]$; $^{59/61}[NaCl^-]$; $^{93/95}[NaCl_2^-]$	3-5
V-rich	$^{51}[V^+]$; $^{67}[VO^+]$; $^{54/56}[Fe^+]$; $^{58/60}[Ni^+]$	sulfate; nitrate; EC	3, 4
Mg-rich	$^{24}[Mg^+]$; $^{41}[MgOH^+]$; $^{54/56}[Fe^+]$; $^{23}[Na^+]$; $^{40}[Ca^+]$; $^{57}[CaOH^+]$	sulfate; nitrate; $^{35/37}[Cl^-]$; $^{26}[CN^-]$; $^{16}[O^-]$; $^{17}[OH^-]$	3-5
Crustal material / Dust	$^{40}[Ca^+]$, $^{56}[Fe^+]$, $^{56}[CaO^+]$	$^{60}[SiO_2^-]$, $^{76}[SiO_3^-]$, $^{79}[PO_3^-]$	3-5

References:

(1) Du, X.; Xie, Q.; Huang, Q.; Li, X.; Yang, J.; Hou, Z.; Wang, J.; Li, X.; Zhou, Z.; Huang, Z.; Gao, W.; Li, L. Development and characterization of a high-performance single-particle aerosol mass spectrometer (HP-SPAMS). *Atmos. Meas. Tech.* **2024**, 17 (3), 1037-1050.

(2) Li, L.; Huang, Z.; Dong, J.; Li, M.; Gao, W.; Nian, H.; Fu, Z.; Zhang, G.; Bi, X.; Cheng, P.; Zhou, Z. Real time bipolar time-of-flight mass spectrometer for analyzing single aerosol particles. *Int. J. Mass Spectrom.* **2011**, 303 (2), 118-124.

(3) Wang, G.; Ruser, H.; Schade, J.; Passig, J.; Adam, T.; Dollinger, G.; Zimmermann, R. Machine learning approaches for automatic classification of single-particle mass spectrometry data. *Atmos. Meas. Tech.* **2024**, 17 (1), 299-313.

(4) Li, L.; Wang, Q.; Zhang, Y.; Liu, S.; Zhang, T.; Wang, S.; Tian, J.; Chen, Y.; Hang Ho, S. S.; Han, Y. Impact of reduced anthropogenic emissions on chemical characteristics of urban aerosol by individual particle analysis. *Chemosphere* **2022**, 303, 135013.

(5) Wang, N.;Zhang, Y.;Li, L.;Wang, H.;Zhao, Y.;Wu, G.;Li, M.;Zhou, Z.;Wang, X. A.-O.;Yu, J. Z. ;Zhou, Y. Ambient particle characteristics by single particle aerosol mass spectrometry at a coastal site in Hong Kong: a case study affected by the sea-land breeze. *PeerJ* **2022**, 10, e14116.

(6) Liu, J.;Peng, J.;Men, Z.;Fang, T.;Zhang, J.;Du, Z.;Zhang, Q.;Wang, T.;Wu, L. ;Mao, H. Brake wear-derived particles: Single-particle mass spectral signatures and real-world emissions. *Environ. Sci. Ecotechnology* **2023**, *15*, 100240.

(7) Kong, Y.; Wang, Q.; Li, L.; Zhang, Y.; Tian, J.; Ma, N.; Zhou, Y.; Liu, H.; Liu, J.; Ran, W.; Liu, J.; Zhu, C.; Han, Y.; Cao, J. Chemical composition and mixing state of elemental carbon-containing particles from solid fuel combustion. *npj Clim. Atmos. Sci.* 2025, 8 (1), 202.

(8) Silva, P. J.; Liu, D.-Y.; Noble, C. A.; Prather, K. A. Size and Chemical Characterization of individual particles resulting from biomass burning of local southern california species. *Environ. Sci. Technol.* **1999**, 33 (18), 3068-3076.

(9) Zhang, Y.; Pei, C.; Zhang, J.; Cheng, C.; Lian, X.; Chen, M.; Huang, B.; Fu, Z.;

Zhou, Z.; Li, M. Detection of polycyclic aromatic hydrocarbons using a high performance-single particle aerosol mass spectrometer. *J. Environ. Sci.* **2023**, 124, 806-822.

(10) Passig, J.; Schade, J.; Oster, M.; Fuchs, M.; Ehlert, S.; Jäger, C.; Sklorz, M.; Zimmermann, R. Aerosol mass spectrometer for simultaneous detection of polyaromatic hydrocarbons and inorganic components from individual particles. *Anal. Chem*. 2017, 89 (12), 6340-6345.

(11) Akhbarizadeh, R.;Dobaradaran, S.;Amouei Torkmahalleh, M.;Saeedi, R.;Aibaghi, R. ;Faraji Ghasemi, F. Suspended fine particulate matter ($PM_{2.5}$), microplastics (MPs), and polycyclic aromatic hydrocarbons (PAHs) in air: Their possible relationships and health implications. *Environ. Res.* **2021**, 192, 110339
